# Supplementary material for: Resolving the full spectrum of human genome variation using Linked-Reads
Source: Genome Res. 2019 Apr;29(4):635–45. doi: 10.1101/gr.234443.118 (PMC6442396; doi:10.1101/gr.234443.118)
Supplement: Supplemental Material [file supp_gr.234443.118_Supplemental_longranger-2.2.zip › longranger-2.2.tar.bz2!/lr2.2-public/lib/stan/cmdstan/stan/doc/api/html/classstan_1_1_vector_view_3_01const_01_t_00_01is__array_00_01throw__if__accessed_01_4.html]

Stan: stan::VectorView< const T, is\_array, throw\_if\_accessed > Class Template Reference


|  |  |
| --- | --- |
|  | Stan  2.5.0  probability, sampling & optimization |


- Main Page
- Related Pages
- Namespaces
- Classes
- Files

- Class List
- Class Index
- Class Hierarchy
- Class Members

All Classes Namespaces Files Functions Variables Typedefs Enumerations Enumerator Friends Macros Pages

- stan
- VectorView< const T, is\_array, throw\_if\_accessed >

Public Types |
Public Member Functions |
List of all members

stan::VectorView< const T, is\_array, throw\_if\_accessed > Class Template Reference

VectorView that has const correctness.
More...

`#include <traits.hpp>`

|  |  |
| --- | --- |
| Public Types | |
| typedef scalar\_type< T >::type | scalar\_t |
|  | |

|  |  |
| --- | --- |
| Public Member Functions | |
|  | VectorView (const scalar\_t &c) |
|  | |
|  | VectorView (const scalar\_t \*x) |
|  | |
|  | VectorView (const std::vector< scalar\_t > &v) |
|  | |
| template<int R, int C> | |
|  | VectorView (const Eigen::Matrix< scalar\_t, R, C > &m) |
|  | |
| const scalar\_t & | operator[] (int i) const |
|  | |

## Detailed Description

### template<typename T, bool is\_array, bool throw\_if\_accessed> class stan::VectorView< const T, is\_array, throw\_if\_accessed >

VectorView that has const correctness.

Definition at line 305 of file traits.hpp.

## Member Typedef Documentation

template<typename T , bool is\_array, bool throw\_if\_accessed>

typedef scalar\_type<T>::type stan::VectorView< const T, is\_array, throw\_if\_accessed >::scalar\_t

Definition at line 307 of file traits.hpp.

## Constructor & Destructor Documentation

template<typename T , bool is\_array, bool throw\_if\_accessed>

|  |  |  |  |  |  |
| --- | --- | --- | --- | --- | --- |
| stan::VectorView< const T, is\_array, throw\_if\_accessed >::VectorView | ( | const scalar\_t & | *c* | ) |  |

inline

Definition at line 309 of file traits.hpp.

template<typename T , bool is\_array, bool throw\_if\_accessed>

|  |  |  |  |  |  |
| --- | --- | --- | --- | --- | --- |
| stan::VectorView< const T, is\_array, throw\_if\_accessed >::VectorView | ( | const scalar\_t \* | *x* | ) |  |

inline

Definition at line 311 of file traits.hpp.

template<typename T , bool is\_array, bool throw\_if\_accessed>

|  |  |  |  |  |  |
| --- | --- | --- | --- | --- | --- |
| stan::VectorView< const T, is\_array, throw\_if\_accessed >::VectorView | ( | const std::vector< scalar\_t > & | *v* | ) |  |

inline

Definition at line 313 of file traits.hpp.

template<typename T , bool is\_array, bool throw\_if\_accessed>

template<int R, int C>

|  |  |  |  |  |  |
| --- | --- | --- | --- | --- | --- |
| stan::VectorView< const T, is\_array, throw\_if\_accessed >::VectorView | ( | const Eigen::Matrix< scalar\_t, R, C > & | *m* | ) |  |

inline

Definition at line 316 of file traits.hpp.

## Member Function Documentation

template<typename T , bool is\_array, bool throw\_if\_accessed>

|  |  |  |  |  |  |
| --- | --- | --- | --- | --- | --- |
| const scalar\_t& stan::VectorView< const T, is\_array, throw\_if\_accessed >::operator[] | ( | int | *i* | ) | const |

inline

Definition at line 318 of file traits.hpp.

---

The documentation for this class was generated from the following file:

- src/stan/meta/traits.hpp


---

[ Stan Home Page ]

*© 2011–2014,
Stan Development Team.*
